# Health Literacy, General Health, and Lifestyle among Infertile Men and Women in the Southeastern Iran

**DOI:** 10.4314/ejhs.v33i5.10

**Published:** 2023-09

**Authors:** Fatemeh Govahi Kakhki, Fatemeh Pakdaman, Nasimeh Khaleghi, Maryam Seraji

**Affiliations:** 1 Student Research Committee, Zahedan University of Medical Sciences, Zahedan, Iran; 2 Department of Health Education and Health Promotion, Zahedan University of Medical Sciences, Zahedan, Iran; 3 Health Promotion Research Center, Zahedan University of Medical Sciences, Zahedan, Iran

**Keywords:** General health, Health literacy, Infertility, Lifestyle

## Abstract

**Background:**

Infertility is a growing social problem, and health literacy is one of the factors that affects infertility, thereby affecting life quality. On the other hand, lifestyle factors exert a considerable impact on reproductive capacity and general health. Against this backdrop, this study aims to determine health literacy, general health, and lifestyle in infertile people in Zahedan, Iran.

**Methods:**

In this descriptive cross-sectional study, 86 male and 181 female participants who referred to Molod Infertility center (AliIbnAbiTalib Hospital) in Zahedan were randomly selected. Health Literacy Standard Questionnaires (HELIA), the General Health Questionnaire (GHQ), and the Miller-Smith Lifestyle Assessment Inventory (LSI) were used to collect data. In addition, data were analyzed by SPSS V.22.

**Results:**

The participants' mean age was 30.87±7.5. Besides, 39% and 70% of the participations did not have enough health literacy and had a moderate lifestyle, respectively. In addition, the general health of 68.9% was exposed to damage. Moreover, there was a significant relationship between the three aforementioned factors, with the participants of higher levels of health literacy having had higher levels of general health and lifestyle.

**Conclusion:**

According to the findings of this study, the infertile people's health literacy was not enough, and most of them had a moderate lifestyle. In addition, their general health was exposed to damage in most cases. Thus, it is necessary to pay attention to providing proper education and health services to infertile men and women to improve their level of health literacy and healthy fertility in order to support childbearing in the society.

## Introduction

Fertility is a key element of reproductive health. On the other side, the World Health Organization (WHO) defines infertility as a global public health issue and the failure to have a pregnancy after 12 months or more of regular unprotected sex (1). According to the WHO, over 1.5 million Iranian couples suffer from infertility (2). Women and men are equally responsible for infertility causes. Most infertility problems are caused by one of these major causes, including semen quality and deficiencies in men and female factors, such as women's diseases of ovulatory dysfunction, the uterus, or tubal-peritoneal diseases(3). Lifestyle is referred to as the inhabitants'characteristics of a region in special places and times, which includes daily behaviors and individuals'functions in activities, jobs, diets, and fun. According to the WHO, 60% of factors associated with the health and quality of life of people are related to lifestyle (4). Furthermore, reproductive health depends on the lifestyle. Various lifestyle dimensions can negatively and positively affect fertility. A study indicated that education in health promotion lifestyle is effective in reducing lifestyle risk factors for infertility and increasing the success rate of assisted reproductive treatment via modifying these risk factors (5). Furthermore, infertility disrupts the affected people's general health and can change people's attitude towards social acceptance and life control (6). Research on infertile couples indicated that infertility had a significant impact on the infertile couples' health status, especially infertile women at the risk of physical symptoms, severe depression, and impaired social functioning (7). The WHO defines health literacy as social and cognitive skills determining individuals' motivation and the ability to understand, access, and use information in the ways that promote and maintain health. Inadequate health literacy affects children, women, and their families (8).

In addition, scientific evidence indicates that people's lifestyle choices and patterns affect their general health in turn (9). Furthermore, health literacy may affect lifestyles. A study showed that people with sufficient health literacy get sick less frequently than others (10). Individuals with adequate health literacy are considered likely to adopt healthy lifestyles (11). Some studies have reported the association between health literacy and healthy lifestyle characteristics (12, 13). A study on the relationship between sexual function, health literacy, and satisfaction among infertile couples showed that most couples'health literacy was borderline, with its adverse effects confirmed on sexual satisfaction, lifestyle, as well as general health(14). In the study of Kilfoyle et al, health literacy played an important role in reproductive knowledge and behavior (15). The importance of couples' awareness of infertility issues has been well established. Besides, the most important approach to decreasing the infertility problem is to try to decrease its occurrence and promote reproductive health. Thus, to prevent the occurrence rate of infertility, having the knowledge of infertility health literacy, general health, and lifestyle is important in each region. Due to cultural, social, and economic differences in Zahedan (Southeastern Iran) and since no study has been conducted in this field, this study tries to investigate health literacy, general health, and lifestyle among infertile men and women in Zahedan City.

## Materials and Methods

**Study design and participants:** This cross-sectional study was conducted on 267 participants (86 men and 181 women) who referred to Molod Infertility Center (Ali Ibn Abi Talib Hospital) in Zahedan, Iran, 2022. Using the sample size formula for mean estimation and the 95% confidence level. The participants were selected via convenience sampling. The inclusion criteria included having a history of infertility for at least one year, not having a history of stressful events in the last 6 months (such as the death of loved ones), and willingness to enter the study.

After receiving the Ethics Committee's Code, data were collected from Molod Centre of Ali ibn AbiTalib Hospital in Zahedan. By briefing the participants on the aim of the study, people would be included in the study if they consented to participate in the research, with an emphasis put on maintaining the confidentiality of their information. People with an incomplete questionnaire or those who were not willing to participate in this study were excluded.

**Demographic information questionnaire:** The Health Literacy Questionnaire (HELIA: Health Literacy for Iranian Adults), the GHQ (General Health Questionnaire), the Miller-Smith lifestyle Assessment Inventory (LSI)questionnaire were used to collect data.

The HELIA Questionnaire was localized by Montazeri, *et al* ,with its reliability and validity confirmed(the Cronbach's alpha coefficient ranging from 0.72 to 0.89)(16). This questionnaire had two parts, including part “a” on demographic characteristics that contained 6 questions (marital status, sex, age, education level, residence place, and occupation)and part “b” with 33 items in the five fields of accessibility (6 items), reading skills (4 items), comprehension (7 items), evaluation (4 items), and decision making (12 items).The questions were ranked as quite easy (5), easy (4), neither easy nor hard (3), hard (2), and quite hard (1). In addition, the maximum and minimum scores of this questionnaire were 165 and 33, respectively. Higher scores indicated higher health literacy.

The GHQ was developed in a few countries in the1960s and 1970s. Goldberg and Williams(1988) reported that the GHQ was translated into about 38 languages, with its validity reported in over 50 studies published(17).The Cronbach's alpha coefficient of this questioner was 0.90(18).The general health questionnaire (with 28 questions) has the four subscales of somatic symptoms (items1-7), anxiety(items 8-14),social dysfunction (items 15-21), and depression(items 22-28),each of which having seven questions. The scoring method in this questionnaire was based on the Likert scale (0, 1, 2, 3).In fact, four possible answers of (1) not at all, (2) not more than usual, (3) rather more than usual, and (4) much more than usual were available for each item. A low score indicated health, and a high score indicated a disorder. A score within the range of 0-21 indicated a very bad condition. Scores within the range of 22- 42 indicated being under threat and damage, scores 43-63 indicated that general health was exposed to damage in many cases, and scores 64-84indicated a serious state of general health.

The LSI questionnaire had 20 questions that were translated from the original questionnaire into Persian and then into English. The validity and reliability of the Persian version of this tool were confirmed by Roohafza et al in 2004.The Cronbach's alpha in this study was 0.864(19).This questionnaire was scored based on the Likert scale, with each question having five answers (always = 1, often = 2, sometimes = 3, rarely = 4, and never = 5). Higher scores indicated an unpleasant and unhealthy lifestyle. On the other side, scores20-45 indicated a poor lifestyle,46-75 indicated a moderate lifestyle, and 76-100 indicated a favorable lifestyle.

**Data analysis:** Data were analyzed by SPSS V.22.Descriptive statistics, including frequency percentage, frequency, standard deviation, and mean were used to describe quantitative variables. After assessing the normality of the data, analyses were performed using the Spearman's correlation coefficient to determine the relationship between health literacy, general health, and lifestyle. To determine the relationship between qualitative demographic variables with health literacy, general health, and lifestyle, an independent-samples t-test and a one-way ANOVA were used.

**Ethical considerations:** This study was approved by the Ethics Committee of the Zahedan University of Medical Sciences under code IR.ZAUMS.REC.1401.152. Informed consent forms were obtained from the participants, and the questionnaires were anonymous.

## Results

A total of 267 participants were enrolled in this study, with the mean age of them having been 30.87±7.5. In fact, 67.8% (n =181) were female,32.2%(n =86) were male, 47.2% (n =126) had under high school education,44.9% (n =120) were unemployed, 15%(n=40) had a smoking history, and 67.4% (n =180) lived in the city (Table 1).

**Table 1:** Frequency distribution of participants' demographic variables and their relationship with health literacy, general health, and lifestyle in infertile participants (n = 267)

Variables	Category	N (%)	Health literacy Mean ± Std. deviation	General health Mean ± Std. deviation	Lifestyle Mean ± Std. deviation
**Gender**	Men	86(32.2)	116±17.69	51±11.8	70.52±10.38
	Women	181(67.8)	115.5±19.41	51.14±10.27	68.98±9.19
	P-value		0.819	0.922	0.221
**Smoking history**	Has it	40(15)	108±17.96	48.47±10.66	69.4±11.47
	Does not have	227(85)	117±18.71	51.55±10.74	69.49±9.26
	P-value		0.005*	0.095	0.955
**Education Level**	Illiterate	24(9)	98±19.74	50.62±9.95	67.04±8.12
	Under high school	126(47.2)	112±17.07	51.19±10.5	69.22±9.4
	University	117(43.8)	123±18.84	51.08±11.27	70.25±10
	P-value		<0.001*	0.973	0.302
**Occupation**	Unemployed	120(44.9)	113±19.27	50.51±10.79	68.25±9.04
	Employee	54(20.2)	123.8±17.59	52.75±9.37	71.48±9.4
	Manual worker	24(9)	111.2±19.48	44.95±10.39	66.83±11.9
	Free job	69(25.8)	115±17.23	52.92±11.19	70.95±9.5
	P-value		0.003*	0.009*	0.05*
**Time of exercise**	Does not have	81(30.3)	112.3±18.55	51.81±10.55	69.35±9.92
	=30 minutes/day	58(21.7)	118.5±22.59	51.79±9.66	70.84±8.43
	< 30 minutes/day	107(40.1)	116.8±16.42	50.15±11.46	67.73±9.39
	> 30 minutes/day	21(7.9)	115.19±19.61	51.14±11.21	75.04±10.35
	P-value		0.230	0.704	0.008*

* P-value≤0.05; based on an Independent-Samples T-Test and a One-Way ANOVA

In addition, the total mean scores of health literacy, general health, and lifestyle were 115.6±18.63, 51±0.77, and 69.5±9.60, respectively, with their minimum scores being 62,15,40 and their maximum score being 165,75,99.

Additionally, 39% of the participants did not have quite sufficient health literacy, general health of 68.9% was exposed to damage in many cases, and 70% had a moderate lifestyle (Table 2).

**Table 2:** Frequency of health literacy, general health, and lifestyle based on score classification (n = 267)

Variables		N(%)	Mean	Std. deviation
Health literacy	Highly adequate	15(5.6)	115.6	18.63
	Adequate	99(37.1)		
	Not quite adequate	104(39)		
	Inadequate	49(18.4)		
General health	Very desirable	2(0.7)	51	10.77
	Under threat and damage	51(19.1)		
	Exposed to damage in many cases	184(68.9)		
	Serious state of general health	30(11.2)		
Lifestyle	Excellent lifestyle	76(28.5)	69.5	9.60
	Moderate lifestyle	187(70)		
	Poor lifestyle	4(1.5)		

Among the individual characteristics, history of smoking, education level, as well as the jobs of infertile men and women had a significant relationship with the health literacy level (p ≤ 0.05). Besides, job was associated with general health (p ≤ 0.05). Additionally, there was a significant relationship between lifestyle and occupations well as duration of exercise (p ≤ 0.05) (Table 1). Based on the results, health literacy had a significant relationship with general health (r = 0.145, p=0.018) and lifestyle (r = 0.326, p<0.001). Accordingly, people with lower health literacy had lower levels of general health and lifestyle. In addition, there was a significant relationship between the two variables of general health and lifestyle (r = 0.347, p<0.001) (Table 3).

**Table 3:** Pearson's coefficient of the relationship between health literacy, health literacy, general health, and lifestyle (n = 267)

Variables	General health	Health literacy	Lifestyle
General health	-	r = 0.145 p=0.018*	r = 0.347 p<0.001*
Health literacy	r = 0.145 p=0.018*	-	r = 0.326 p<0.001*
Lifestyle	r = 0.347 p<0.001*	r = 0.326 p<0.001*	-

* Correlation is significant at the level of 0.05.

## Discussion

This study aimed to investigate health literacy, general health, and lifestyle in infertile men and women who referred to Molod Infertility center (Ali Ibn AbiTalib Hospital) in Zahedan.

The findings of this study indicated that health literacy had a statistically significant relationship with the variables of education level, smoking history, and occupation. Our findings were consistent with those of several other reports (8, 20, 21). Employed people seem to have higher levels of health literacy than housewives; this is due to the fact that they generally have more updated information and social network support as well as more academic education than housewives. Moreover, in this study, like two other studies (20, 22), individuals with high levels of education had higher average health literacy. Therefore, high levels of education play an important role in enhancing health literacy. As this study and another study showed (23), there was a significant relationship between health literacy and smoking. In fact, smoking was a risk factor for psychological distress among infertile individuals. On the one hand, in our study, like another study, job was positively associated with general health (24).

Our results also showed that lifestyle was associated with the variables of occupation and exercise. These findings were consistent with two other studies (25, 26). In fact, employed people with higher education levels and more facilities attach more importance to their health than other people and choose a healthier lifestyle. In addition, evidence shows that there is a positive relationship between lifestyle and exercise, being consistent with the present study. In fact, exercise increases the chances of having conception. Regular moderate exercise improves fertility potentials as well as lifestyle in infertile subjects (27).

In this investigation, health literacy levels were not quite sufficient in the participants. Some past studies confirmed this result (28, 29), while others reported inconsistent results (30, 31). These different results could be due to the dependence of health literacy on cultural conditions and socioeconomic factors, being the most important factors. It should be noted that there were few studies on health literacy and infertility.

Social factors can influence infertility. According to our findings, the participants' general health was exposed to damage. On the other hand, in our study being consistent with Simionescu's study, infertile couples suffered more stress than normal and healthy ones, having been at a higher risk of developing mental disorders. This could have been caused by insufficient psychological assistance, ineffective treatment, low socioeconomic status(such as unemployment, low income, etc.), and non-assistance on the part of one's partner (24).

Another result from this study was that the average lifestyle in most of the participants was in line with Mirghafourv's study on infertile couples(32, 33), yet it was inconsistent with another study in Spain (19). This difference could have been due to different levels of education, age, or facilities available, which could have led to differences in responses to questions.

In addition, the present study showed a positive relationship between health literacy and general health. This finding was consistent with those of previous studies (34, 35). In fact, people of insufficient health literacy exhibited more unpredictable behaviors than those with health literacy. In contrast, a report by Qanbari, *et al* indicated that no significant association existed between general health and health literacy levels (31). This difference might have been due to differences in the study group, sample size, and the tools used for data collection.

In general, the results of this research showed a significant statistical association between lifestyle and health literacy levels of infertile people. This finding was consistent with those of previous studies (36, 37).

A health-promoting lifestyle focuses on lifestyle promotion via mental development, physical activity, health responsibility, nutrition, stress management, and interpersonal relationships. These factors exert some effects on infertility among people. Thus, to have healthy fertility, have children, and support youths in a population, it is necessary to develop interventional educational programs for subjects on health literacy, lifestyle, and general health. Such a program is likely to result in health promotion, wellbeing, self-actualization, confidence, satisfaction, and better feelings among populations. It seems that paying attention to this type of lifestyle may include general healthy behaviors throughout one's life (2). In addition, another study showed that health literacy levels were associated with mental, physical, and environmental aspects, yet they were not associated with lifestyle (38). The reason for this inconsistency could have been due to the tool used to measure the quality of life.

Health literacy can affect the use of information, sexual performance, and the solving of infertility problems among couples, thereby exerting a positive effect on infertile people' lifestyle (37). Health literacy is a determining factor in the health of women and children, which affects the health of society. In fact, improving health literacy and transferring clinical knowledge to understandable models for policymakers, healthcare providers, pregnant women, and their spouses will play an effective role in reducing health risks during pregnancy and solving problems among infertile couples in future.

One of the limitations of this study was that it was a self-report in which the answers and information on psychological conditions of the participants were received at the time of answering the questions. Besides, as the current study was cross-sectional, it is possible to better understand causal relationships between the variables by conducting research in a longer span of time.

The results of this investigation indicated that health literacy and lifestyle were not quite sufficient, moderate respectively, and the general health in infertile participants were exposed to damage. In fact, people who were employed had better health literacy, lifestyle, and general health than others. Employment makes a person able to interact effectively and avoid unfavorable responses. In addition, it indicates the social and behavioral health of a person. In fact, there was a significant correlation between health literacy, lifestyle, and general health. In the end, it is recommended that programs and interventions be developed for infertile men and women to increase their levels of health literacy, general health, lifestyle, health fertility, and childbearing, especially in deprived areas.
